# Radiomics in prostate cancer imaging for a personalized treatment approach - current aspects of methodology and a systematic review on validated studies

**DOI:** 10.7150/thno.61207

**Published:** 2021-07-06

**Authors:** Simon K.B. Spohn, Alisa S. Bettermann, Fabian Bamberg, Matthias Benndorf, Michael Mix, Nils H. Nicolay, Tobias Fechter, Tobias Hölscher, Radu Grosu, Arturo Chiti, Anca L. Grosu, Constantinos Zamboglou

**Affiliations:** 1 Department of Radiation Oncology, Medical Center - University of Freiburg, Faculty of Medicine. University of Freiburg, Germany; 2 German Cancer Consortium (DKTK). Partner Site Freiburg, Germany; 3 Department of Radiology, Medical Center - University of Freiburg, Faculty of Medicine. University of Freiburg, Germany; 4 Department of Nuclear Medicine, Medical Center - University of Freiburg, Faculty of Medicine. University of Freiburg, Germany; 5 Department of Radiation Oncology - Division of Medical Physics, Medical Center - University of Freiburg, Faculty of Medicine. University of Freiburg, Germany; 6 Radiotherapy and Radiation Oncology, Faculty of Medicine and University Hospital Carl Gustav Carus, Technische Universität Dresden.; 7 OncoRay-National Center for Radiation Research in Oncology, Faculty of Medicine and University Hospital Carl Gustav Carus, Technische Universität Dresden, Helmholtz-Zentrum Dresden-Rossendorf, Dresden, Germany; 8 Institute of Computer Engineering, Vienne University of Technology, Vienna, Austria; 9 Department of Biomedical Sciences, Humanitas University, Via Rita Levi Montalcini 4, 20090 Pieve Emanuele - Milan, Italy; 10 IRCCS Humanitas Research Hospital, Via Manzoni 56, 20089 Rozzano - Milan, Italy; 11 Berta-Ottenstein-Programme, Faculty of Medicine, University of Freiburg, Germany; 12 German Oncology Center, European University of Cyprus, Limassol, Cyprus

## Abstract

Prostate cancer (PCa) is one of the most frequently diagnosed malignancies of men in the world. Due to a variety of treatment options in different risk groups, proper diagnostic and risk stratification is pivotal in treatment of PCa. The development of precise medical imaging procedures simultaneously to improvements in big data analysis has led to the establishment of radiomics - a computer-based method of extracting and analyzing image features quantitatively. This approach bears the potential to assess and improve PCa detection, tissue characterization and clinical outcome prediction. This article gives an overview on the current aspects of methodology and systematically reviews available literature on radiomics in PCa patients, showing its potential for personalized therapy approaches. The qualitative synthesis includes all imaging modalities and focuses on validated studies, putting forward future directions.

## Introduction

In global cancer statistics of men prostate cancer (PCa) is the most frequently diagnosed malignancies in the world and the fifth leading cause of death worldwide 1,2. Therefore, the development of accurate diagnostic tools is of great importance. Many modern imaging modalities provide a great value in screening, diagnosis, treatment response measurement and prognosis evaluation of PCa patients. A suspicious digital rectal examination and/or an elevation of prostate specific antigen (PSA) in blood serum lead to transrectal ultrasound (TRUS) guided biopsy for histopathologic verification of PCa 3. In recent years an augmented approach of this strategy, including magnetic resonance imaging (MRI), has gained traction in clinical application and was incorporated into guidelines 4. MRI is not only employed prior to biopsy, but for local staging and follow up 5. Nevertheless, diagnostic accuracy is still hampered by inter-observer variability and exactness of lesion detection does not seem to be warranted 6-8. In an attempt to improve interpretation, reporting and acquisition standards for global harmonization “Prostate Imaging - Reporting and Data System Version 2.1” (PI-RADSv2.1) were established 9. Bone scans and computer tomography (CT) used to be standard of care (SoC) for staging and re-staging. As of late prostate specific membrane antigen positron emission tomography (PSMA-PET) has been implemented into clinical practice as recommended in current guidelines for staging and restaging 10-13. Additionally, growing evidence proclaims the use of PSMA-PET for intraprostatic lesion detection and segmentation 14-18. In addition to an accurate diagnosis, proper and decisive risk stratification is crucial, due to a variety of treatment options in different clinical scenarios. However, recommended models for risk classification 5,19,20 might not always predict the final outcome in every disease stage of PCa 21,22. Thus, new concepts for adequate detection and risk stratification towards precision medicine and personalized treatment are required. With the rise of big data analysis, the computer-based extraction of pre-defined image features in terms of “hand-crafted” radiomic features (RF) is an emerging field in research that might satisfy this need. It is hypothesized that medical images contain more information than discernible visually by trained professionals. Simplified these RF might provide more information about a tumor or other tissues facilitating diagnosis, risk stratification and therapeutic outcome. The advantage of radiomics is the utilization of SoC images without additional required effort and the abundance of medical images available, which can be utilized for longitudinal monitoring. Another benefit is that radiomics examines whole tumors as opposed to biopsy schedules which are prone to sampling errors due to intratumoral heterogeneity 23,24. Thereby, radiomics offer great potential for personalization of therapeutic approaches, in particular for image-based disciplines such as radiation oncology. This review gives an overview of methodological aspects of radiomics firstly, followed by the methodology of our literature search and a qualitative synthesis of radiomics in prostate cancer subdivided by imaging modalities and based on a systematic search. There have been reviews on this topic but mostly focusing on MRI whereas our review includes all imaging modalities and concentrates on papers with internal or external validation 25-29.

## Current Methodological Aspects of Radiomic Feature Extraction

### Radiomics Pipeline

**Figure 1:**
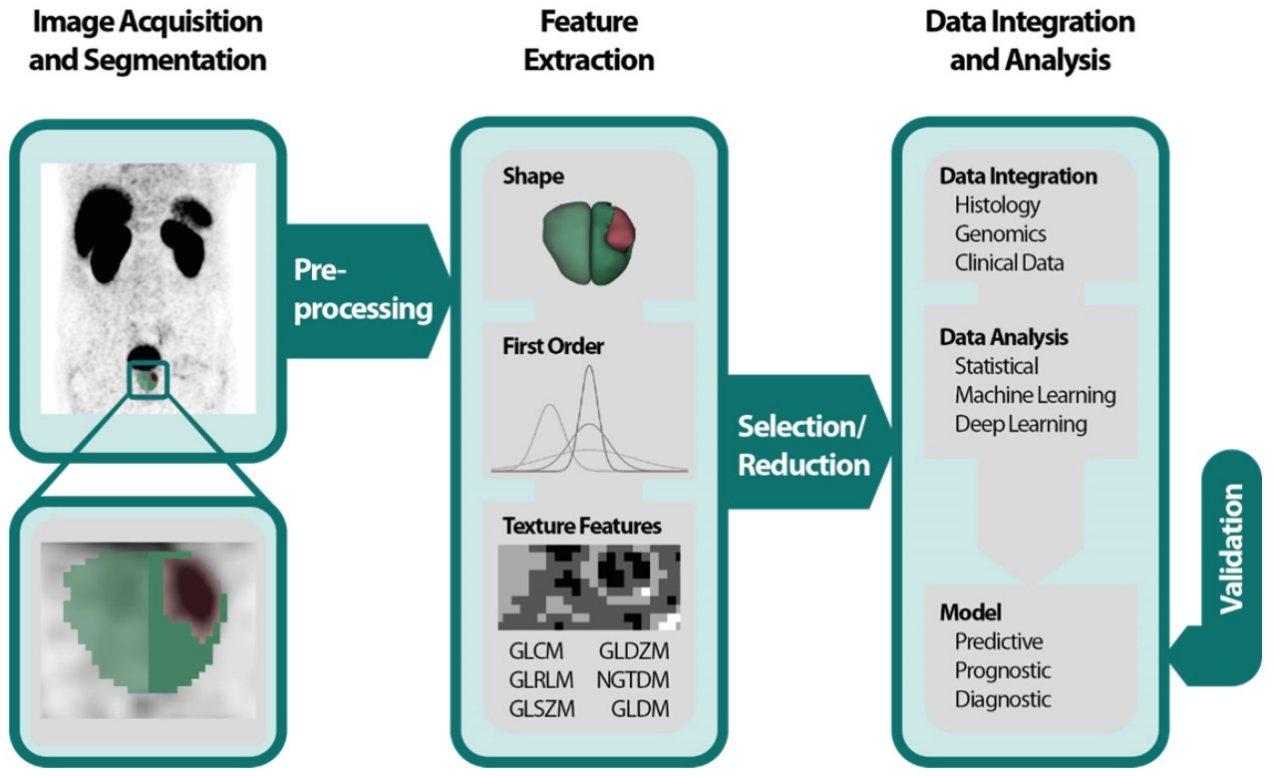
Radiomics pipeline depicts the data processing and operations to build a radiomics model with validation. First an image is acquired and segmented manually, semiautomatically or fully automatically. Then feature extraction is performed after preprocessing. Feature classes are shape features, first order features and texture features. Due to the abundance of RF a selection or reduction should be performed before or while integrating with histology, genomics or clinical data. Data analysis can be performed by using classical statistical models, with machine learning or deep learning. A predictive, prognostic or diagnostic model is built and should be internally or externally validated. Abbreviations: GLCM=gray level co-occurrence matrix; GLDZM = gray level distance zone matrix; GLRLM = gray level run length matrix; GLSZM = gray level size zone matrix; NGTDM = neighboring gray tone difference matrix; NGLDM = neighboring gray level dependence matrix; RF= Radiomic features.

The Radiomics Pipeline (Figure 1) is the entire sequence of data processing from imaging to a diagnostic, predictive or prognostic model based on RF. It is subdivided into three major operations.

(1) Image acquisition and preprocessing

(2) High-throughput feature extraction

(3) Data integration and data analysis

The Image Biomarker Standardization Initiative (IBSI) published a reference manual to harmonize the feature extraction by providing (i) definitions, (ii) a standardization of the radiomics pipeline, (iii) reference datasets and (iv) a reporting scheme 30.

#### (1) Image acquisition and preprocessing

All imaging modalities mentioned in the introduction can be utilized for PCa radiomics: TRUS, MRI, PSMA-PET, CT and bone scan. It is important to mention that heterogeneities in image acquisition und image reconstruction algorithms due to different local standards are culpable for missing repeatability and reproducibility of RF 31-34. Prospective trails with fixed imaging protocols could aim to ensure that a scan yields similar results in the same patient when repeated on the same system i.e. repeatability as well as on different systems and institutes i.e. reproducibility 33. After image acquisition the volume of interest (VOI) is delineated manually, semi-automatically or fully automatically. If manual segmentation is performed a sophisticated protocol should be used throughout the whole dataset to minimize inter-observer variability 35. Subsequently and before feature extraction images should be pre-processed, *e.g.*, by intensity inhomogeneity correction or noise filtering for MR-images 32. It is known that preprocessing sequences can also have a significant impact on the robustness and reproducibility of RF and identification of generalizable and consistent preprocessing algorithms is a pivotal step 36.

#### (2) High-throughput feature extraction

The spatial and gray level information of the segmented voxels is used in numerous mathematical calculations to extract pre-defined “hand-crafted” RF. They can be computed with various open-source packages like PyRadiomics 37, IBEX 38, RaCat 39, QIFE 40, MaZda 41, CERR 42 or LIFEx 43 as well as commercial products 44. Additionally, radiomic codes implemented in MatLab ® are commonly used. It is very important to validate the used software tools, especially homemade software, with datasets provided by the IBSI 30, to increase reproducibility, robustness and comparability across different platforms. Current versions of PyRadiomics, LIFEx, RaCat, CERR and adapted version of IBEX comply with IBSI. Besides the extraction of hand-crafted RF, convolutional neuronal networks as a subfield of machine learning (ML) can be used for pattern recognition and image feature analyses by applying the actual images 45. This can be done in combination with predefined “hand-crafted” features 46,47, but mostly ML algorithms engineer models, based on large amounts of data, autonomously 48.

#### (3) Data integration and data analysis

Often a vast number of RF are computed, and the abundance of RF demands feature selection and/or reduction to avoid overfitting and to exclude not relevant or redundant features. Many features are correlated with each other; these redundant features might be depicted with heatmaps and should be omitted 49,50. Additionally, ML algorithms like minimum redundancy and maximum relevance or fisher score can be used to assess the correlation between RF 51,52. Other options for feature reduction are prioritizing robust features 34. An overview of feature reduction steps including quantitative comparisons of performance is given by Leger et al 53 and Parmar and colleagues 54. The analysis of the remaining features can be conducted by using the RF alone or in combination with other clinical parameters by applying classical statistical methods or ML for data integration and modelling. Examples of ML classifiers are random forests, support vector machines and nearest neighbors for instance 51,55. To avoid overfitting, it is recommended to control false positive results by correcting for multiple testing when the data analysis is based on classical statistical methods 56,57. After the generation of a model based on RF, validation should be executed to evaluate its performance and to assess generalizability 58. In recent years ever more emphasis has been laid on this last step 33-35. During an internal validation, the data is usually split in 3 datasets: (i) one training dataset for optimizing the parameters of a model, (ii) one validation dataset for hyperparameter optimization *e.g.*, the depth of a tree or a deep-learning architecture and (iii) one test set for the final assessment. The latter might be used independently for validation. During cross-validation (CV) usually small datasets are divided accordingly in an iterative process. K-fold CV partitions the dataset in k subsets using one as a validation and the rest for training. This process is repeated for each subset. Leave-one-out CV operates similarly but leaves one patient for validation while using the rest for training CV should be used with caution especially with leave-one-out CV tending to be overly optimistic 59,60. External validation based on independent datasets from different institutes enables the highest quality of validation 58.

### Hand-crafted Radiomic Features

“Hand-crafted” RF 48 can be grouped in shape descriptors, 1^st^ order features and texture features. Shape features describe the morphology of the VOI for instance the size, volume or diameter. 1^st^ Order features are based on an intensity histogram derived of the segmented voxels 24. Texture features are more advanced and do not only rely on voxel intensities i.e., gray levels but on spatial information as well. First introduced by Haralick *et al.* the gray level co-occurrence matrix (GLCM) assesses the gray levels of pairs of neighboring voxels 61. Others like the gray level size zone matrix (GLSZM) 62 and the gray level run length matrix (GLRLM) 63 analyze groups of consecutive voxels, zones, or runs of connected voxels in one direction, respectively. For a more complete description of features, texture matrices and their mathematical calculations we recommend the IBSI and their documentation 30. High-order features are calculated on filter transformed images like wavelets or gaussian bandpass filer 24.

## Methodology

**Figure 2:**
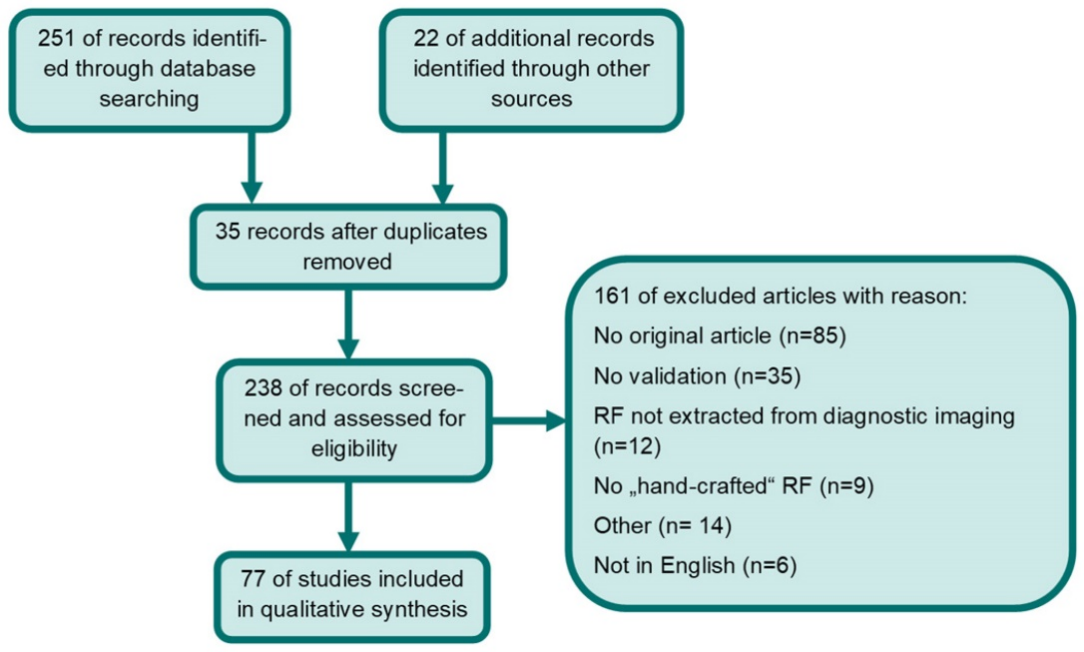
Flow diagram of systematic database search and records excluded. Abbreviations: RF= radiomic features

Studies eligible for inclusion complied with the following criteria: articles had to be on PCa radiomics with predefined “hand-crafted” features derived from MRI, TRUS, CT, Choline- or PSMA-PET and needed to apply internal or external validation. Excluded were papers not written in English and non-original articles. Two of the co-authors (SKBS and ASB) performed independently a PubMed/Medline, EMBASE and Cochrane Library database search for the terms: (cancer of prostate[MeSH Terms]) AND ((texture features) OR (radiomics)). If the two independent readers included or excluded studies differently a third reader (CZ) decided on eligibility. This was performed in 11 cases. The time period considered in this literature review was from 1^st^ of January 2014 64 to 1^st^ of January 2021. 251 articles were located. Additionally, 22 manuscripts were identified through other sources (*e.g.*, google scholar or references in screened manuscripts). 35 duplicates were removed. Only articles that met inclusion criteria were included. Finally, 77 studies were included in the qualitative synthesis. Please see Figure 2 for a detailed description of the performed literature search according to PRISMA 65. Due to heterogeneity of imaging modalities, in the applied Radiomics pipeline and the analyzed endpoints no quantitative analysis was performed. Additionally, we assessed whether the utilized software complied with IBSI.

Furthermore, ongoing clinical trials were screened on “clinicaltrials.gov”. Studies eligible for inclusion fulfilled the following criteria: ongoing trials on PCa RFs with “hand-crafted” features derived of MRI, TRUS, CT or PSMA-PET. Trials with unknown status were excluded. CZ performed the search for the terms (“Condition or disease: prostate cancer” AND “radiomicsl” OR “texture features”). Six trials were located, and one trial (NCT03294122) was excluded due to unknown trial status.

## Results

### I MRI

Literature research revealed 57 original papers computing RF on multiparametric magnetic resonance tomography (mpMRI) imaging (see Table 1). The most common segmented VOI was intraprostatic tumor (n = 48) 66-114, of which five studies focused on tumor location in transitional zone (TZ) 72,73,81,95,112 and five studies in peripheral zone (PZ) 68,71,76,82,95. 14 studies selected the prostate as VOI 69,71,73,78,82,87,96,97,104,110,112, 115-118 of which one study analyzed the prostate excluding urethra and intraprostatic dominant lesions 97 and one study differentiated between different prostate zones 115. Four studies considered PCa localization in fused histopathologic information as VOI 75,119-121 and one study the area of biopsy including the surrounding 15 mm 114. One study included peritumoral areas 96. The rectal wall was delineated as VOI in one study 122. RFs were extracted from T2-weighted images (T2w, n = 52) 66-76,78-91,93-107,109-113,116-122, apparent diffusion coefficient (ADC, n = 38) 67,70-72,74,77-81,83-85,87,89-96,98-100,103-105,108-113,115,116,120,122, diffusion weighted imaging (DWI, n = 21) 66,69-73,76,77,80,82,86,88,90,94,98,105,107,112,115-117, dynamic contrast enhanced (DCE, n = 13) 66,73, 79,80,82,89,91,104-107,114,120, computed high-b DWI (CHB-DWI) (n = 5) 69,74,75,100,115, T1-weighted sequences (T1w, n = 3) 72,106,117 and diffusion tensor imaging (n = 1) 76, respectively. PCa detection (n = 19) 66-82,114,115, Gleason Score (GS) discrimination or upgrading from biopsy to prostatectomy (n = 22) 83-100,116,119-121, extracapsular extension (n = 5) 90,103-105,120, biochemical recurrence (n=4) 108-110,117, segmentation (n = 3) 112, 113, 118, bone metastasis (n = 2) 106,107, treatment response (n = 1) 111, and rectal toxicity (n = 1) were investigated as primary endpoints 122. Most of the studies adopted internal validation (n = 50) 66,67,69-72,74-95,97,99,100,103-107,109,111-117,119-122 utilizing ML algorithms like CV (n = 26) and/or leave-one-patient-out CV (n = 10) 66,67,69,72,74,75,81,83,84,86,88,90-94,97,99,103,104,111,113-115,119, 122, as well as independent internal validation cohorts (n = 24) 70,71,76-80,82,85,87,89,90, 95,100,105-107,109,112,116,117,120,121. External validation was performed in seven studies 68,73,96, 98,108,110,118. Eleven studies complied with IBSI 71,78-80,94,97,103,105,108,109,118.

In two preliminary studies, Cameron *et al.* developed a model based on mpMRI RF for PCa detection implementing a comprehensible identification scheme by grouping features into the categories morphology, asymmetry, physiology and size (MAPS) 66, 67. The model had an accuracy of 87% and outperformed models based on conventional mpMRI features 66. Furthermore Khalvati *et al.* proposed a RF based framework for PCa detection and localization 74. Additional studies, among which is the externally validated study by Viswanath *et al.* (ROC-AUC 0.683-0.768 across 3 sites), showed good area under the receiver operating characteristics curve (ROC-AUC) for PCa detection 68-70,75. Five studies considered the tumor location (TZ vs. PZ) for RF based PCa detection 71-73,76,81. Two of these demonstrated improved PZ and TZ lesion classification with ADC based RF 71 and a high ROC-AUC value of 0.86 72. Gholizadeh *et al.* developed a framework of combined T2w, DWI and DTI features for differentiation of PCa and non-PCa voxels 76. Bleker *et al.* demonstrated that the addition of DCE-RFs does not improve performance of T2w and DWI-RF based models to detect clinically significant PCa in the PZ 80. A multi-institutional and externally validated study by Ginsburg *et al.* showed lower results for a PZ specific classifier for PCa detection with ROC-AUC of 0.6-0.71 73.

GS prediction and discrimination were assessed in 22 studies 83-100,116,119-121. Most of these studies showed that RF models can differentiate between GS groups (low-, intermediate- and high risk), to predict GS or to predict GS upgrading between the biopsy and radical prostatectomy 83-91,93,114,119,120. Chaddad *et al.* introduced novel RF based on Joint Intensity Matrix to predict GS (ROC-AUC 0.64-0.82 depending on GS groups) 84. A study from Rozenberg *et al.* however, could not show that ADC features were predictive for GS upgrading in intermediate-risk prostate cancer 92. Penzias *et al.* demonstrated that RF and quantitative histomorphometry correlate and are predictive for GS 121. Hou *et al.* investigated the prediction of clinically significant PCa (GS≥7) in PIRADS 3 lesions, which could be a useful tool for biopsy guidance 94. Zhang and colleagues showed that a radiomic signature, consisting of 10 features, identified clinically significantly PCa (GS ≥ 3+4) with AUC values of 0.95 (training), 0.86 (internal validation), and 0.81 (external validation) 84. Algohary *et al.* reported that RFs from T2w and DWI sequences are associated with clinically significant PCa, being even more relevant than PIRADSv2 evaluation in some patients 99. In an external validated study, a combination of intra- and peritumoral RF resulted in AUCs of 0.87 and 0.75 for the differentiation of low risk PCa versus high-risk PCa or intermediate- and high risk PCa defined by D'Amico Risk Classification 82.

Five studies investigated RF for the prediction of extracapsular extension and reported high AUC values between 0.80-0.90 for radiomic signatures based on T2w and ADC sequences 90,103,120 that outperformed clinical or nomogram models 104,105. Two studies from Wang *et al.* and Zhang *et al.* showed that mpMRI derived RFs show good performance for bone metastasis prediction in untreated PCa with an ROC-AUC up to 0.92 85,107. Six studies analyzed the performance of RF in terms of outcome 108-111,117,122. Bourbonne *et al.* externally validated an ADC based RF (SZE_GLSZM_), which was identified in a previous study 94 for biochemical recurrence (BCR) prediction after surgery with an accuracy of 0.76 108,109. Shiradkar *et al.* demonstrated a ML classifier derived from T2w and ADC RF with good prediction of BCR after surgery or RT. which was externally validated with a AUC of 0.73 110. Another RF model by Zhong *et al.* showed good performance for BCR prediction after RT of localized PCa 117. Abdollahi and colleagues indicated that RF from pre- and post-treatment ADC images are predictive in terms of treatment response after primary external beam radiotherapy 111. Another study from this group demonstrated that RF of pre-radiotherapy images provided good ROC-AUC values of up to 0.81 for rectal toxicity prediction 122. One study by Sunoqrot *et al.* elaborated a quality system to asses automated prostate segmentations with external validation 118 and two studies from Lay *et al.* and Giannini *et al.* addressed RF-based PCa segmentation 112,113.

### II PSMA-PET

Literature research revealed five original papers 123-127 using PET images to extract RF (see Table 2). Four studies used intraprostatic tumor as VOI for RF extraction 124-127 and one study non-PCa tissue in PET 123. One study performed external validation of their results, the remaining studies were internally validated by CV 124,125 or two independent cohorts 126,127. Four of these studies complied with IBSI 123-126.

Three studies aimed for GS discrimination 124-126 and demonstrated excellent ROC-AUC values between 0.81-0.91. Two studies chose intraprostatic tumor detection as study endpoint 123,126. A study by Zambolgou *et al.* reported two distinct RFs (SAE, local binary pattern small-area emphasis; SZNUN, local binary pattern size-zone non-uniformity normalized) with good performance to detect significant PCa lesions not visible in PSMA-PET/CT. This result was externally validated by an independent cohort 123. Cysouw *et al.* demonstrated a RF based machine learning model to predict lymph node involvement, presence of metastases, GS prediction (≥8) and presence of extracapsular extension 125.

### III Other imaging modalities

Literature research revealed six original papers using CT scans 128-133 and four using TRUS imaging (n = 4) 47,134-136 to extract RF (see Table 3). The respective VOI for RF extraction were prostatic gland (n = 6) 47, 128, 129, 133-135, intraprostatic tumor (n=5) 136, bone metastases (n = 1) 132, lymph node metastasis (n=1) 131 and rectal/bladder wall (n = 1) 130. All studies were internally validated by CV (n = 8) 128-134,136 or two independent cohorts (n = 3) 47,131,135. Only one study complied with IBSI 131.

GS discrimination by RF was the aim of four studies using TRUS 134, CT 128,133 or CBCT images 129 and reported excellent ROC-AUC values between 0.77-0.98 including one or multiple RF for modeling. Three studies defined intraprostatic tumor detection in TRUS images 47,134,136 as a study endpoint. Again, the implementations of one or multiple RF led to very promising results in PCa detection. The study of Wu *et al.* implemented RF for automatic prostate gland delineation in TRUS images 135 and observed similar results compared to manual delineation by experts. One study 130 implemented RF to predict bladder and bowel toxicity after radiotherapy of PCa patients and reported ROC-AUCs of up to 0.77 by integrating clinical information with RF. The study of Osman *et al.* suggested that RF derived from CT images might enhance interpretation of treatment response of bone metastases 133 and Acar *et al.* demonstrated that RFs derived from CT images of PSMA-PET/CT scans could accurately distinguish between metastatic lesions and sclerotic area 132. The RF model in a study by Peeken *et al.* outperformed conventional measures for detection of lymph nodes metastases 131.

### IV Ongoing trials

In total, 5 studies were identified using mpMR imaging (n = 4), PET (n = 1), CT (n = 1) and bone scans (n = 1) to extract RF (see Table 4). Four studies evaluate RF for outcome prediction during or after several treatment approaches: active surveillance, surgery, radiotherapy, or radionuclide therapy in addition to chemotherapy. Two of those four studies integrate RF with molecular markers for modelling. One study evaluates whether RF extracted from lesions describe histologic characteristics, lymph node involvement and extension.

## Discussion

PCa radiomics is an emerging research field with a high potential to offer non-invasive and longitudinal biomarkers for personalized medicine. In our review based on a qualitative synthesis of 77 studies, most papers address MRI based RFs, which is not surprising since MRI is the actual SoC for primary PCa staging. Other imaging modalities such as CT, PSMA-PET, TRUS and bone scan are less commonly used, but their application has improved in the recent years. This trend might proceed with the increased usage of PSMA PET/CT for staging of primary, recurrent, and metastasized PCa patients. One major focus of the included papers was PCa detection. Keeping in mind that image interpretation and segmentation is hampered by interobserver variability 6,137 implementation of RF might enhance diagnostic performance. Advances in automated segmentation of intraprostatic tumor lesions, for example by deep learning-based approaches such as convolutional neural networks, might overcome this limitation 138.

The other focus is GS discrimination, reflecting the need for improvements in risk stratification. It is not surprising that most of the studies chose GS discrimination, since GS is the most established histologic biomarker. In clinical routine, the GS before primary PCa therapy is evaluated in tissue cores obtained by biopsy. However, due to intratumoral heterogeneity the GS in biopsy cores and prostatectomy specimen is discordant in 20-60% of the patients 139,140. Nevertheless, the bioptic GS has a significant impact on clinical management as it defines the patient's risk group influencing for example the duration of androgen deprivation therapy or the dose to the prostate during radiation therapy 141. RF-based GS prediction might account for intratumoral heterogeneity leading to over- or underestimation of the GS in biopsy specimen. For instance, Zamboglou *et al.* demonstrated that a PSMA PET-derived RF (QSZHGE) may outperform biopsy mapping for GS 7 vs ≥8 discrimination 126. Recently, Chu *et al.* examined the PSMA expression in a combined cohort of more than 18 000 radical prostatectomy specimens and observed a correlation between PSMA expression and the GS 142. This finding provides a strong biological rationale for non-invasive GS prediction based on RF extracted from PSMA PET images.

However, several studies proposed that a thorough analysis of PCa tissue characteristics (*e.g.* by genomic analyses) might outperform GS for risk prediction 143. Radiogenomics combines RF analysis with genomic information thus linking both research fields. Our literature search revealed five studies but none of the studies were internally or externally validated and thus excluded. Nevertheless, they should be mentioned, highlighting this modern and innovative approach 25,144-147. A pilot study by Sun *et al.* showed weak correlations between RF and hypoxia gene expressions, providing an opportunity to assess the hypoxia status in PCa 146. Two studies by McCann *et al.* and Switlyk *et al.* demonstrated an association between RF and the genetic marker phosphatase and tensin homolog 144,147. Stoyanova *et al.* identified radiomic signatures which reflected genes that are over- and underexpressed in aggressive prostate cancer 25. Additionally, another study with a small patient cohort by Kesch *et al.* suggests that RF signatures could distinguish between lesions of different aggressiveness 145.

Direct prediction of treatment outcome with RF is investigated in ongoing clinical trials especially. A possible explanation for this finding is the long follow-up time needed to provide reliable clinical information of treatment outcomes in PCa patients. Just a few manuscripts (n = 24) address extraprostatic extension (n = 6), BCR (n = 6), segmentation (n = 4), bone metastasis (n = 3), lymph node detection (n = 3) and radiotherapy toxicity (n = 2). Considering that most PCa patients are long-term survivors after treatment a reliable prediction of toxicity is warranted. Due to the lack of predictive models for toxicity prediction, we consider this field of major interest for future studies. Some of the excluded studies featured interesting concepts for the use of radiomics and treatment associated toxicity in PCa patients. Radiotherapy toxicity prediction was investigated for femoral head fractures 148 and urethral strictures after high-dose rate brachytherapy 149. One paper used RF for response assessment of PCa bone lesions derived of and ADC maps 150. Rossi *et al.* did not compute RF on imaging but on rectum and bladder 3D dose-volume histogram distributions. This add-on improved the prediction of late toxicities after radiotherapy 151. These extensive fields of application demonstrate the great potential of radiomics and its clinical implementation from diagnosis to outcome and toxicity prediction in an era of big data and individualized medicine.

Overall, most of the included studies presented good to high AUC values. However, these findings need to be considered diligently regarding publication bias and the variability observed in RF. As illustrated above the radiomic pipeline is a sequence of operations and each operation can be modified 31. RF and models are sensitive to those modifications and consequently, investigations on RF variability, robustness and reproducibility are demanded 31.

Texture features are increasingly sensitive to acquisition parameters with growing spatial resolution 152 as well as reconstruction algorithms 153. Yang et al proposed a simulation framework to asses robustness and accuracy of radiomic textural features with different MRI acquisition parameters and reconstruction algorithms 153. Recently Rai *et al.* developed a 3D printable phantom to measure repeatability and reproducibility of MRI-based radiomic features which could facilitate multi-center studies to harmonize image protocols and thereby tackling some of these challenges 160.

Multiple segmentations can reduce variability and bias in RF extraction of manually, semiautomatically or automatically segmented VOIs 154. To increase robustness of segmentation manual methods should be avoided. In PET images, Bashir et al demonstrated that semiautomatic threshold-based methods yield superior interobserver reproducibility 155. Additionally, CNN based segmentation methods showed good performance 156.

Isaakson et al investigated normalization techniques to enhance comparability across different subjects and visits 158. Scalco *et al.* investigated different generally adopted image intensity normalization techniques for T2w-MRI images and demonstrated a relevant impact on reproducibility of RFs 154.

Schwier *et al.* investigated the variability of RF in MRI by using different filters, normalization, and image discretization techniques and observed that RF were sensitive to these pre-processing procedures. Hence, they recommended detailed reporting of the pre-processing steps and the use of open-source software 29. Orlhac *et al.* reported that ComBat harmonization is efficient and enables MRI data pooling from different scanners and centers 155.

Two studies investigated repeatability of MRI-derived RFs and concluded that repeatability of many RFs is moderate and that a set of reproducible image features is desirable 156, 157. Delgadillo *et al.* investigated repeatability of RF derived from CBCTs and reported that only five radiomic features were repeatable in < 97% of the reconstruction and preprocessing methods 159. Bologna *et al.* proposed an approach to assess RF stability without multiple acquisitions and segmentations that could be used for preliminary RF selection. In addition, the authors advocated that RF derived of ADC maps behave differently based on the region extracted *e.g.* RF derived from head and neck tumors are less stable than those derived of sarcomas 161. Pfaehler *et al.* recommends to investigate the repeatability of RF for every tumor type as well and for every PET-Tracer 30.

These papers demonstrate the fragility of RFs and the need of reproducible RF sets in order to enable a broad clinical application.

Consequentially, more research on prostate MRI and PSMA-PET RF robustness should be performed. Other approaches to tackle RF variability is the standardization of RF definitions and calculations which IBSI tries to promote 28. The radiomics quality score, a tool to evaluate methodologic quality of radiomic studies, could also be used 32. With higher quality, evidence on RF robustness like the recent metanalysis of Zwanenburg *et al.* pitfalls could be uncovered and described 33. These methodological aspects seem all the more important, since only a few studies identified in this review are explicitly IBSI compliant and future work needs to focus on this issue. We furthermore encountered problems to validate the studies IBSI compliance, since most studies don't give sufficient information about the used software and calculations of RF. We therefore plead for uniform and detailed specifications.

Nevertheless, validation is pivotal considering the variability of RF. 35 of 238 articles were excluded due to missing validation. In internal validation different types might be utilized like the aforementioned ML algorithms, k-fold CV or leave-one-out CV, as well as independent datasets for model development and validation. A proper methodology and the separation of training and validation dataset is demanded at all times 157. Our synthesis detected 64 articles with internal validation (k-fold CV n = 36; leave-one-out CV n = 11, two cohorts n = 29). 14 studies used more than one validation type. External validation is the gold standard and was performed in eight of the identified articles. Only one manuscript reported about external validation of an already published model 108. These findings put ever more emphasis on the validation of radiomics models especially externally and from already published models 58.

Many studies used ML for model building and verification. ML and deep learning as a subfield are emerging and harbor great potential 48. Li *et al.* used deep learning in combination with “hand-crafted” features and has successfully applied it in differentiating unilateral breast cancer from low-risk patients 46. Segmentation of PCa lesions by deep learning networks is explored without “hand-crafted” features 158.

This review focusses on the clinical aspects of RF demonstrating its great potential to affect management of PCa. However, some technical aspects have not been further investigated: information on the used algorithms for RF extraction or ML approaches were not provided. Additionally, we did not state whether the published models or the parameters are publicly available.

In conclusion, most research in PCa radiomics focuses on PCa detection and GS discrimination. MRI as SoC is the most used imaging modality for RF computation for now, but PSMA-PET is gaining evidence in a wide variety of clinical settings. Most of the results suggest good to high performance of radiomics models but should be considered carefully due to RF variability. Further research is demanded on RF sensitivity and robustness especially on RF extracted of prostate MRI and PSMA-PET.

## Figures and Tables

**Table 1:** List of included articles on RFs derived of MRI. In the second column # are the number of patients enrolled retrospectively (R) or prospectively (P). In the fourth column the volume of interest (VOI) is presented accompanied by the type of segmentation in brackets M = manual, SA = semiautomatic and A = fully automatic. The last column contains information on validation. “e” stands for external validation and “I” for internal. The number stands for the number of cohorts used. 2 means one for development and one for testing.

Prostate cancer detection						
Study	#	Imaging Modality	VOI (Segmentation)	Endpoint(s)	Results	Validation
Cameron *et al.* 67	5 (R)	T2w, ADC	PCa (A +M)	PCa detection	RF model outperformed conventional mpMRI feature models.	i (LOO)
Cameron *et al.* 66	13 (R)	T2w, DWI, DCE	PCa (A)	Classifiers for PCa detection	RF model outperformed conventional mpMRI feature models.	i (CV, LOO)
Viswanath *et al.* 68	85 (R)	T2w	PCa, PZ, central gland (M)	Classifier for voxel-wise PCa detection	Boosted Decision Tree classifier has the highest ROC-AUC for detecting PCa., Boosted Quadratic-Discriminant Analysis is the most accurate and robust in detection of PCa extent across three sites. The ground truth was established by whole mount histology.	e (CV with external centers)
Khalvati *et al.* 69	20 (R )	T2w, DWI, CDI, CHB-DWI	PCa, prostate (M)	Classifier for PCa detection	Support vector machine classifier improved PCa auto-detection.	i (LOO)
Xu *et al.* 70	331 (R)	T2w, DWI, ADC	PCa	Benign vs. malignant lesions	BpMRI improved discrimination between benign and malignant lesions.	i (2)
Bonekamp *et al.* 71	316 (R)	T2w, DWI and ADC	PCa, PZ, prostate (M)	PCa ISUP ≥2	Quantitative ADC measurement improves differentiation of benign vs malignant lesions, ML comparable, performance of zone-specific models was lower.	i (2+CV)
Sidhu *et al.* 72	76 (R)	T1w, T2w, DWI, ADC	PCa in TZ (M)	PCa detection in TZ	TZ derived RF can discriminate TZ-PCa.	i (LOO)
Ginsburg *et al.* 73	80 (R)	T2w, DWI, DCE	PCa, TZ, prostate (M)	PCa detection in TZ and PZ	TZ-specific classifier significantly improves accuracy of PZ-PCa detection.	e (3 institutions)
Parra *et al.*^114^	52 (R)	DCE	Habitat = biopsy +15 mm (M)	PCa detection (significant)	Habitats from DCE predict clinically significant PCa well.	i (LOO)
Khalvati *et al.* 74	30 (R)	T2w, ADC, CHB-DW, CDI	PCa (A)	Framework for PCa detection	Proposed framework (MPCaD can be utilized to detect and localize PCa.	i (LOO)
Wang *et al.* 75	54 (R)	T2w, CHB-DW	PCa, histological-radiological correlation (M)	Classifier for PCa detection (significant)	SVM classifier improves performance of PI-RADS v2 for clinically relevant PCa.	i (LOO)
Gholizadeh *et al.*^76^	16 (P)	T2WI, DWI, DTI	PCa in PZ(M)	Differentiation pf PCa and non-PCa	Voxel‐based supervised machine learning models generated a binary classification of cancer probability maps.	i (2+LOO)
Hu *et al.* 77	136 (P)	DWI, ADC	PCa (M)	PCa detection	A mixed model based on the clinically independent risk factors and mp-MRI radiomics score showed the best performance.	i (2)
Woźnicki *et al.*^78^	191 (R)	T2w, ADC	PCa + Prostate (M)	PCa detection and clinical significance	An ensemble machine learning model combining radiomics, PI-RADS, prostate specific antigen density and digital rectal examination resulted in a good predictive performance.	i (2+CV)
Qi *et al.* 79	199 (R)	T2w, ADC, DCE	PCa (M)	PCa prediction on patients with PSA level of 4-10ng/ml	The combined model incorporating all sequences, age, PSA density and the PI‐RADS v2 score yielded good performance for prediction of PCa.	i (2)
Dulhanty *et al.* 115	101 (R)	ADC, CHB-DWI	Prostate zones (M)	PCa detection based on 10 anatomical zones	Zone-level radiomic sequences distinguish between positive and negative zones.	i (CV)
Bleker *et al.* 80	206 (P)	T2w, DWI, ADC, DCE	PCa (SA)	csPCa in PZ	Addition of DCE-RFs does not improve performance of T2w- and DWI-RF based models. Multivariate RF selection with extreme gradient boosting outperformed univariate selection.	i (2)
Wu *et al.*^81^	90 (R)	T2w, ADC	PCa in TZ (M)	Differentiation of PCa inTZ	Proposed models using quantitative ADC, shape and texture features, show good performance for TZ PCa detection and remained accurate when comparing TZ PCa with stromal BPH and in smaller lesions.	i (CV)
Kwon *et al.* 82	344 (R	T2w, DCE, DWI, proton density-weighted	Prostate, PZ, PCa (M)	Detection of csPCA Classification methods	Random forest classification showed the highest AUC.	i (2)
Gleason score						
Hectors *et al.*^83^	64 (R)	T2w, ADC, diffusion kurtosis imaging maps	PCa (unknown)	Aggressiveness (GS, Gene expression, Decipher)	14 RF with significant correlation to GS, 40 DWI features with significant correlation to Gene expression, ML models with excellent performance to predict Decipher score ≥ 6.	i (CV)
Chaddad *et al.* 85	99 (R)	T2w, ADC	PCa (M)	GS grouping (6/3+4/4+3)	Joint Intensity Matrix-derived RF (n=5) are independent predictors of GS.	i (2)
Chaddad *et al.* 84	99 (R)	T2w, ADC	PCa (A)	GS grouping (6/3+4/4+3)	T2w and ADC derived RF can predict GS.	i (CV)
Sun *et al.* 119	30 (R)	T2w	PCa on histology (M)	GS, Risk groups	ADC, GLCM and GLRLM discriminate between high grade and low grade PCa. The combination further improved AUC.	i (CV)
Jensen *et al.* 86	112 (R)	T2w, DWI	PCa (M)	GS, risk group	Zonal-specific DWI and T2w derived RF differentiate between PCa lesions of all GS.	i (LOO + CV)
Chen *et al.* 87	381 (R)	ADC, T2w	PCa, prostate(M)	PCa/non-PCa, high grade GS /low grade GS 6 compared to PI-RADSv2	T2w and ADC RF show high efficacy in distinguishing PCa vs non-PCa and high-grade vs low-grade PCa.	i (2)
Toivonen *et al.* 88	62 (R)	T2w, DWI, T2-mapping	PCa	GS	T2w and DWI derived RF show good classification performance for GS of PCa.	i (LPOCV + CV)
Zhang *et al.* 89	166 (R)	T2w, ADC, DCE	PCa (M)	PCa upgrading	T2w, ADC and DCE derived RF can predict GS upgrading from biopsy to radical prostatectomy.	i (2)
Min *et al.* 90	280 (R)	T2w, DWI, ADC	PCa (M)	PCa detection (significant)	MpMRI derived RF discriminate between GS 3+4 or lower.	I (CV)
Li *et al.* 91	63 (R)	T2w, ADC, DCE	PCa (M)	GS in CG PCa	Support vector machine classification achieves accurate GS classification of PCa in central gland.	i (CV)
Rozenberg *et al.* 92	54 (R)	ADC	PCa (M)	Prediction of GS upgrading and Differentiation of GS 3+4 and 4+3	ADC derived texture features are not predictive of GS upgrading after radical prostatectomy.	i (CV)
McGarry *et al.* 120	48 (P)	T2w, ADC, DCE	PCa on histology (M)	Gleason probability maps	RF based mapping successfully stratifies high- and low-risk PCa.	i (2)
Penzias *et al.* 121	36 (R)	T2w	PCa on histology (M)	GS, risk group, correlation with QH	RF and quantitative histomorphometry features correlated with these RF are predictive for of GS.	i (2)
Fehr *et al.* 93	217 (R)	T2w, ADC	PCa (M)	GS risk group differentiation	Automatic classifiers achieve accurate classification of GS.	i (CV)
Hou *et al.* 94	263 (R)	T2w, DWI, ADC	PCa, (M)	Clincially significant PCa (GS≥7) in PIRADS 3 lesions	Radiomics ML model of all sequences has potential to predict csPCa in PIRADS 3 lesions to guide biopsy.	i (CV)
Li *et al.* 95	381 (R)	T2w, ADC	PCa in TZ and PZ (M)	Clincically significant PCa	Radiomics model can predict csPCa with high accuracy (AUC ≥-98).	i (2)
Gong *et al.*^116^	489 (R)	T2w, DWI, ADC	Prostate	Identification of high grade PCa (>GS7)	DWI RF-model and combination of T2w and DWI achieved high accuracy in prediction of GS >7.	i (2, CV)
Algohary *et al.* 96	231 (R)	T2w, ADC	PCa lesion, peritumoral area (M)	Differentiation of PCa Risk Groups according to D'Amico	Combination of peritumoral and intratumoral RFs improved the risk stratification results by 3-6% compared to intra-tumoral features alone.	e (2)
Gugliandolo *et al.* 97	65 (R)	T2w	Prostate excluding urethra and dominant intraprostatic lesions (M)	Prediction of GS, PIRADS v2 Score and Risk Group	Radiomic signature consisting of the combination of 3D GLCM and intensity domain category features were able to discriminate between low- and intermediate-grade malignancy.	i (CV, LOO)
Zhang *et al.* 98	159 (R)	T2w, DWI, ADC	PCa (M)	Discrimintation of csPCa and clincially insignificant PCa	A radiomic signature of 10 features, was significantly associated with csPCa. A nomogram of this signature and ADC values showed even better AUCs.	e (2, CV)
Algohary *et al.* 99	56 (R)	T2w, ADC	PCa (M)	Prediction of csPCa in active surveillance patients	7 T2w-based and 3 ADC-based RF exhibited statistically significant differences between malignant and normal regions in the training groups. The 3 constructed ML models yielded good accuracy	i (CV)
Abraham *et al.* 100	162 (R)	T2w, ADC, high B-Value Diffusion-Weighted (BVAL)	PCa (A)	Classification of Grade Groups	The novel method using texture features and stacked sparse autoencoder was able to classify PCa grade groups moderately.	i (2, CV)
Extracapsular extension						
Ma *et al.* 120	119 (R)	T2w	PCa (M)	ECE of PCa	T2w derived RF predict side specific ECE.	i (2)
Ma *et al.* 90	210 (R)	T2w	PCa (M)	ECE prior to RP	T2w derived RF outperformed radiologist in predicting ECE.	i (2)
Stanzione *et al.* 103	39(R)	T2w, AdC	PCa index Lesions (M)	Classifier for ECE prediction	Bayesian Network was the best classifier for ECE prediction.	i (CV)
Losnegard *et al.* 104	228 (R)	T2w, ADC, DCE	Prostate, PCa (M+A)	ECE Prediction in high and unfav. Intermediate risk PCa	12 RF extracted from manual segmentation combined with a Random Forest classifier can predict ECE with an AUC of 0.74. Features from T2W and ADC showed a good performance. A combined model performed even better.	i (CV)
Xu *et al.* 105	95 (R)	T2w, DWI, ADC, DCE	PCa (M)	ECE	8 RF were used to build a radiomics model with an AUC of 0.92. A radiomics nomogram with clinical features yielded similar results.	i (2)
Bone metastasis						
Wang *et al.* 106	176 (R)	T2w, DCE T1w	PCa (M)	Bone metastasis prediction	T2w and DCE derived RF were predictors for BM.	i (2)
Zhang *et al.* 107	116 (R)	T2, DWI, DCE	PCa (M)	Prediction of bone metastasis in newly diagnosed PCa	The radiomics nomogram based on 11 RFs and clinical risk factors, showed good performance to promote individualized prediction of bone metastasis.	i (2)
Biochemical recurrence						
Bourbonne *et al.* 109	107 (R)	T2w, ADC	PCa (SA)	Prediction of BCR and biochemical relapse free survival after RP in high risk PCa	One ADC derived RF (SZE_GLSZM_) was predictive for BCR and bRFS (AUC 0.76).	i (2)
Bourbonne *et al.* 108	195 (R)	ADC	PCa (SA)	BCR	External validation of the identified ADC derived RF (SZE_GLSZM_) for BCR and bRFS prediction after RP.	e (2)
Shiradka *et al.* 110	120 (R)	T2w and ADc	PCa, prostate (M)	BCR after RP or RT	BpMRI RF-trained machine learning classifier can be predictive of BCR.	e (2)
Zhong *et al.*^117^	91 (R)	T1w, T2w, DWI	Prostate (M)	BRC of localized PCa after RT and neoadjuvant endocrine therapy.	MRI derived RFs can predict BCR after RT with good performance.	i (2, CV)
Treatment response						
Abdollahi *et al.* 111	33 (P)	T2w, ADC, pre- and post IMRT	PCa (M)	Therapy response (RT), GS, T-stage	T2w and ADC derived RF and ML correlate with IMRT response.	i (CV)
Toxicity						
Abdollahi *et al.* 122	33 (P)	T2w, ADC	Rectal wall (M)	Rectal toxicity	Pre-IMRT MRI RF predict rectal toxicity.	i (CV)
Segmentation						
Sunoqrot *et al.* 118	635 (R)	T2w	Prostate gland (M)	Quality System for automated prostate segmentation	Proposal of a quality check for automated segmentation of the prostate in T2W MR image.	e (2, CV)
Lay *et al.* 112	224 (R)	T2w, ADC, DWI	PCa (M) Prostate and TZ (A)	PCa segmentation	Random forest sampling strategy and instance-level weighting improve PCa detection performance compared to support vector machine.	i (2, CV)
Giannini *et al.* 113	58 (R)	T2w, ADC	PCa (M)	PCa segmentation	Proposed method with GLCM texture features computed on ADC and T2w images reduced the number of false positives and increased the precision of PCa detection.	i (CV)

Abbreviations: ADC=Apparent diffusion coefficient, BCR=biochemical recurrence, bpMRI=biparametric magnetic resonance imaging, bRFS=biochemical recurrence free survival, CDI=current density imaging, csPCa= clinically significant prostate cancer, CV=cross validation, DCE=dynamic contrast enhanced, DTI= diffusion. tensor imaging, DWI=diffusion weighted imaging, GLCM= gray level co-occurrence matrix, GLRLM=grey-level run length matrix, GS=Gleason score, IMRT=intensity modulated radiotherapy, LOO=leave one out, LPOCV=leave-pair-out cross-validation, M=manual confirmation, ML=machine learning, mpMRI=multiparametric magnetic resonance imaging, PCa=Prostate cancer; PZ=peripheral zone, RF=radiomic feature, ROC-AUC=are under the receiver operating characteristics curve, RP=radical prostatectomy, T1w= T1-weighted imaging, T2w=T2-weighted imaging, TZ= transitional zone.

**Table 2:** List of included articles on RFs derived from PSMA-PET images. In the second column # are the number of patients enrolled retrospectively (R) or prospectively (P). In the fourth column the volume of interest (VOI) is presented accompanied by the type of segmentation in brackets M = manual, SA = semiautomatic and A = fully automatic. The last column contains information on validation. The number stands for the number of cohorts used. 2 means one for development and one for testing.

Study	#	Imaging Modality	VOI (Segmentation)	Endpoint(s)	Results	Validation
Zamboglou *et al.* 123	20 (P) 52 (R)	[68Ga]Ga-PSMA-11 PET	Non-PCa tissue	Visually not-detected lesions	2 distinct RF with good performance (SAE, SZNUN)	e (2)
Papp *et al.*^124^	52 (P)	[^18^F]FMC/ [^68^Ga]Ga-PSMA-11 PET/MRI	PCa (M)	Risk group discrimination, BCR	Machine learning RF based models.	i (CV)
Cysouw *et al.*^125^	76 (P)	[^18^F]DCFPyL PET	PCa (SA)	Lymph node metastasis, metastasis, GS≥ 8, extracapsular extension	Radiomics-based machine learning models.	i (CV)
Zamboglou *et al.* 126	60 (R)	PSMA-PET	PCa (M) on PET images and on co-registered histology	PCa detection, GS, pN1 status	QSZHGE: quantization algorithm + short zones high gray-level emphasis.	i (2)
Alongi *et al.* 127	46 (R)	18F-Choline PET	PCa (unknown)	PCa patients' outcome	13 selected RF.	i (2)

Abbreviations: CV= cross-validation; PCa = prostate cancer; GS = Gleason score, SAE, local binary pattern small-area emphasis; SZNUN, local binary pattern size-zone non-uniformity QSZHGE= quantization algorithm + short zones high gray-level emphasis.

**Table 3:** List of included articles on RFs derived from other imaging modalities than MRI. In the second column # are the number of patients enrolled retrospectively (R) or prospectively (P). In the fourth column the volume of interest (VOI) is presented accompanied by the type of segmentation in brackets M = manual, SA = semiautomatic and A = fully automatic. The last column contains information on validation. “e” stands for external validation and “I” for internal. The number stands for the number of cohorts used. 2 means one cohort for development and one for testing.

Study	#	Imaging Modality	VOI (Segmentation)	Endpoint(s)	Results	Validation
**Prostate**						
Zhang *et al.* 47	113 (R)	TRUS: B-mode, Sono- elastography	Prostate (M)	PCa detection	Multimodal feature (4 RFs) learning.	i (2)
Wildboer *et al.* 134.	50 (R)	TRUS: B-mode, contrast enhanced US	Prostate (A)	PCa detection, GS	Multiparametric classifier (n=14).	i (CV)
Wu *et al.* 135	132 & 5 videos (R)	TRUS: B-mode	Prostate (A&M)	Prostate segmentation	Prostate segmentation framework utilizing speckle-induced texture features.	i (2)
Huang *et al.* 136	342 (R)	TRUS (M)	Rectangle around the biopsy core	PCa detection	RF for a support vector machine classifier.	i (CV)
Osman *et al.* 133	342 (R)	CT	Prostate (M)	GS, risk group discrimination	Radiomics classifier.	i (2, CV)
Tanadini-Lang *et al.* 128	41 (R)	CT perfusion	Prostate (M)	GS, risk group discrimination	Single and combined use of RF and conventional CT perfusion parameters.	i (CV)
Bosetti *et al.* 129	31 (R)	Cone-beam CT	Prostate (M)	Tumor stage, GS, PSA level, risk group discrimination, BCR	Histogram-based Energy and Kurtosis and a shape-based feature predict BCR and high risk.	i (CV)
**Toxicity**						
Mostafei *et al.* 130	64 (P)	CT	Pre-treatment Rectal-& Bladder wall (M)	RT toxicity GI/GU ≥ grade 1 CTCAEv4.03	Cystitis: clinical-radiomics (n=4) model. Proctitis: radiomics (n=3) model.	i (CV)
**Lymph nodes**						
Peeken *et al.* 131	80 (R)	Contrast-enhanced CT from PSMA PET/CT scans	Lymph nodes (M)	Lymph node metastasis	Radiomics model significantly outperformed all conventional CT parameters.	i (CV, 2)
**Bone metastases**						
Acar *et al.* 132	75 (R)	CT from PSMA PET/CT	Bone metastases	Discrimination of bone metastases that responded after treatment	Weighted k-nearest neighborhood algorithm.	i (CV)

Abbreviations: CT = computed tomography, CV= cross-validation, GI=gastrointestinal, GS = Gleason score, GU=genitourinary; PCa = prostate cancer; QSZHGE= quantization algorithm + short zones high gray-level emphasis, TRUS = Transrectal Ultrasound

**Table 4:** List of identified ongoing trials to extract radiomic features. Only aims concerning radiomics are mentioned above. In the second column # are the number of patients enrolled retrospectively (R) or prospectively (P). The third column displays the imaging modality (mpMRI=multiparametric magnetic resonance imaging, PSMA/FDG-PET=prostate specific membrane antigen fluorodeoxyglucose positron emission tomography, CT=computer tomography). The fourth column gives an overview of the study's aim(s).

Study	#	Imaging Modality	Aim(s)
NCT03979573	90 (P)	mpMRI	Identification and monitoring of patients with RF in combination with clinical and molecular markers during active surveillance of PCa to reduce discontinuation.
NCT02242773	207 (P)	mpMRI	Correlation of RF with progression during active surveillance and with genomic signatures and other biomarkers.
NCT03180398	20 (P)	mpMRI	Extracted RF are used to identify dominant lesions within the prostate. These RF are monitored longitudinally to analyze their correlation with the local control.
NCT04219059	200 (R)	mpMRI	Evaluates if RF on primitive prostate lesions can describe histological characteristics, lymph node involvement and disease extension.
NCT04343885	140 (P)	PSMA/FDG-PET, CT, bone scans	Prognostic and predictive value RF from PET, CT or bone scans after Lutetium-177 PSMA radionuclide treatment and/ or chemotherapy.
